# How mobile phone addiction is associated with suicidal ideation in university students in China: Roles of depression and online social support

**DOI:** 10.3389/fpsyg.2022.1001280

**Published:** 2022-12-23

**Authors:** Huahua Hu, Xue Yang, Phoenix K. H. Mo, Chengjia Zhao, Beibei Kuang, Guohua Zhang, Guangyao Lin

**Affiliations:** ^1^School of Public Health and Primary Care, The Chinese University of Hong Kong, Shatin, China; ^2^The Chinese University of Hong Kong Shenzhen Research Institute, Shenzhen, China; ^3^Department of Psychology, School of Mental Health, Wenzhou Medical University, Wenzhou, China; ^4^College of International Relation, National University of Defense Technology, Nanjing, China; ^5^The Affiliated Kangning Hospital of Wenzhou Medical University, Wenzhou, China

**Keywords:** mobile phone addiction, suicidal ideation, depression, online social support, Chinese university students

## Abstract

**Background:**

Recent studies have highlighted mobile phone addiction (MPA) as a potential risk of suicidal ideation. However, the mechanisms underlying that association require attention.

**Objective:**

This investigation aims to examine whether the relationship between MPA and suicidal ideation would be mediated by depression, and buffered by online social support (OSS) in university students.

**Methods:**

A convenient sample of 1,042 Chinese university students completed the measures of mobile phone addiction, depression, suicidal ideation, OSS in classroom settings. Moderated mediation analyses were performed to test the roles of depression and OSS in the association between MPA and suicidal ideation.

**Results:**

MPA was positively associated with suicidal ideation through depression (*indirect effect* =. 23, 95% CI: 0.18, 0.28, *p <* 0.001); OSS moderated the association between depression and suicidal ideation (*B* = − 0.09, 95% CI: −0.13, −0.04, *p < 0*.001). Specifically, the effect of depression on suicidal ideation was weaker in individuals with high (versus low) OSS. OSS moderated the association between MPA and suicidal ideation (*B* = 0.06, 95% CI: 0.02, 0.10, *p =* 0.001). The influence of MPA on suicidal ideation was non-significant among individuals with high OSS but negatively significant among students with low OSS.

**Conclusion:**

The results enrich the understanding of how MPA may increase suicidal ideation, and highlight the potential importance of reducing depression and enhancing OSS to prevent suicidal ideation in university students.

## Introduction

The usages of mobile phones are prevalent and have become an important part of people’s lives, especially among young people, including university students. They bring convenience and enjoyment with multiple functions, maintain social connection, and help to improve interpersonal relationships and quality of life (Aljomaa et al., 2016). However, mobile phone addiction (MPA) occurs when individuals are overindulging in mobile phone use and accompanied with withdrawal symptoms, dependence, tolerance, and social functioning issues (Choi et al., 2015). The prevalence of MPA (e.g., Malaysia, China, and Saudi Arabia) varies from 14 to 48% among university students from different countries (Aljomaa et al., 2016; Liang and Leung, 2018; Lei et al., 2020). MPA has been regarded as an emerging mental health issue, as it may pose risks of health issues, such as sleep disorders (Sahin et al., 2013; Chen et al., 2016), depressive symptoms (Jun, 2016), and suicidal ideation (Chen et al., 2020).

Suicidal ideation is another health issue in young people which refers to thoughts about ending one’s life (CDC, 2011), which occurs before suicide attempts in the suicide process. Suicide has become the second dominant source of global death in youth (WHO, 2019). For each suicide, there are more than 20 suicide attempts (WHO, 2019). Suicide ideation and attempts are the leading indicators of deaths by suicide (Klonsky et al., 2016). Studies from various countries (e.g., Indonesia, Saudi Arabia, China, United Kingdom, and the United States) have found that between 9 and 37% of young adults have thought of suicide in the past year (Bruffaerts et al., 2019; Eskin et al., 2019; Wang et al., 2019; Akram et al., 2020). According to the ideation to action framework, the development of suicide ideation and suicide attempts include various personal and interpersonal predictors and complicated mechanisms (Klonsky and May, 2014). To reduce suicide attempts and the corresponding deaths and disability in young people, it is imperative to unveil the risk and protective factors of suicidal ideation. Prior studies have mainly focused on adolescents (Wang et al., 2014; Chen et al., 2020). For example, a recent study found that MPA was associated with suicidal ideation through depression among adolescents (Chen et al., 2020). More attention and efforts are needed to university students, considering the high prevalence of MPA and suicidal ideation in this group (Li et al., 2014; Chen et al., 2016).

This study aims to investigate the relationship between MPA and suicidal ideation in university students, also looking for potential mediators and moderators of this association. The compensatory internet use model, the strain theory of suicide and empirical studies have suggested that MPA, as a maladaptive coping strategy (Kardefelt-Winther, 2014; Garcia-Oliva and Piqueras, 2016; Zhang, 2019), may be associated with a series of mental health issues, such as depression and suicidal ideation (e.g., Steinbuechel et al., 2018; Ong and Thompson, 2019; Sohn et al., 2019). According to stress coping theory and three-step theory of suicide (Lazarus and Folkman, 1984; Klonsky and May, 2015), MPA may be associated with depression, which in turn, associated with suicidal ideation. The social support buffering hypothesis (Cohen and Wills, 1985) and empirical evidence indicates that online social support can provide an important buffer for risk factors and finally prevent depression and suicidal ideation (e.g., Harter and Leahy, 2001; Liu et al., 2017). Therefore, we propose a moderated mediation model in which the relationship between MPA and suicidal ideation would be mediated by depression, and this mediation would be moderated by online social support.

### MPA and suicidal ideation

MPA may be a significant factor influencing suicidal ideation. According to the model of compensatory internet use and the classical characteristics (e.g., escape, tolerance) of MPA, MPA can be a maladaptive coping used for self-distraction or escape from negative feelings or problems (Kardefelt-Winther, 2014; Garcia-Oliva and Piqueras, 2016). The strain theory of suicide and empirical evidence suggests that inadequate coping skills in problem solving (with negative life events) can be a strain which induces frustration and further lead to suicidal ideation (Najmi et al., 2007; Ong and Thompson, 2019; Zhang, 2019). It is, therefore, conjectured that MPA may also give rise to suicidal ideation. Both cross-sectional and longitudinal studies supported the positive association between internet-related addictions (e.g., Facebook Addiction Disorder) and suicidal ideation (Wang et al., 2014; Brailovskaia et al., 2020; Chen et al., 2020). A systematic review revealed that suicidal ideation had stronger positive associations with internet addiction among teens and young adults than suicidal actions (Steinbuechel et al., 2018). Another systematic review concluded that young people with suicidal ideation were frequent users of online forums (Daine et al., 2013). Therefore, we assume that MPA would be positively correlated with suicidal ideation (H1).

### The mediation role of depression

Depression may play a mediation role in the relationship between MPA and suicidal ideation. Theoretically, it can be supported by the stress coping theory and the three-step theory of suicide (Lazarus and Folkman, 1984; Klonsky and May, 2015). First, MPA may lead to depression. According to the stress coping theory (Lazarus and Folkman, 1984), negative coping with stress may result in decreased well-being. Since MPA can be seen as a negative coping strategy (Kardefelt-Winther, 2014) and leaves problems and negative emotions unsolved, this in turn may result in depressive symptoms (Seiffge-Krenke and Klessinger, 2000; Evans et al., 2015). Cross-sectional research (Evans et al., 2015; Chen et al., 2016; Gao et al., 2017) and longitudinal studies (Coyne et al., 2019; Zhang et al., 2020) support the association between MPA and depression. Furthermore, a meta-analysis study demonstrated that problematic mobile phone usage could predict depression among young people (OR = 3.17; 95% CI = [2.30, 4.37]; Sohn et al., 2019). Hence, it is expected that MPA would be associated with depression (H2).

In turn, depression may increase suicidal ideation. The three-step theory of suicide, a theory within ideation-to-action framework, suggests that depression is a strong predictor of suicidal ideation as it can increase psychological pain, hopelessness, and disconnectedness (Klonsky and May, 2015). Their relationship has been well documented in a number of longitudinal and meta-analysis studies (e.g., Ribeiro et al., 2018; Gili et al., 2019; Soto-Sanz et al., 2019; Hayes et al., 2020). Therefore, we assume that depression would be associated with suicidal ideation (H3).

To our best knowledge, no empirical study has tested the mediation effect of depression in the relationship between MPA and suicidal ideation among university students. However, several cross-sectional studies have displayed that depression mediated the association between problematic/excessive use of mobile phone and suicidal ideation in adolescents (Chen et al., 2020), and between MPA and quality of life (Gao et al., 2017) among university students. Retrospective studies of adolescents and adults showed that depressive symptoms/depression mediated the relationship between some risk factors (e.g., pain, sleep disturbances) and suicidal ideation (Lewcun et al., 2018; Richardson et al., 2018). Given these indirect findings, we assume that depression would be a crucial mediator in the correlation between MPA and suicidal ideation among university students (H4).

### The moderation role of online social support

Interpersonal factors, such as social support, may moderate the proposed mediation model. Social support has been portrayed as support relationships that can help individuals to cope with stress, and keep healthy (House et al., 1988). The social support buffering hypothesis (Cohen and Wills, 1985) proposes that social support can provide psychological and instrumental coping resources to reduce potential harms and negative consequences of negative stimuli, and thus benefit well-being. Social support carries signal of social acceptance and connection, which may lead to reconstruction of positive self-perception that prevent depression and suicidal ideation (Harter and Leahy, 2001; Cole et al., 2017). It is therefore a critical buffer for risk factors. Numerous empirical studies have established the buffering role of social support between a range of risk factors (e.g., intrusive thoughts and community violence victimization) and health outcomes (e.g., anxiety and depressed mood; Haden and Scarpa, 2008; Escalera et al., 2019). In addition, online social support (OSS) is the support obtained through online settings, and has started to draw more research attention (Liang and Wei, 2008). Thus, it is expected that online social support would moderate the relationships between MPA and depression, between MPA and suicidal ideation. Specifically, the effect of MPA on depression would be weaker among individuals with higher (compared to lower) levels of OSS (H5). Similarly, the effect of MPA on suicidal ideation would be weaker among individuals with higher (compared to lower) levels of OSS (H6).

Three-step theory of suicide also suggests that while psychological pains and hopelessness are predictive to suicidal ideation, connection with others and the world can be protective for people experiencing depressive symptoms such as psychological pain and hopelessness (Klonsky and May, 2015). The pervasive use of mobile phone, with the multiple social networking apps and functions, provides a media for people to enhance and maintain social connection, improve relationship quality and reduce social isolation (Lewcun et al., 2018; Hayes et al., 2020). OSS might be particularly important to university students as they may move to another city for study and thus maintain their previous social networks (e.g., families, high school classmates) online. Thus, OSS may buffer the impacts of threat such as depression on suicidal ideation by providing coping resources, emotional support, information support, and instrumental support for individuals to cope with negative stimuli. We assume that OSS would moderate the relationship between depression and suicidal ideation. Specifically, the effect of depression on suicidal ideation would be weaker among individuals with higher (compared to lower) levels of OSS (H7).

Empirical studies on OSS are limited and have reported inconsistent results. Some studies argued that, unlike general social support, OSS may sometimes lead to adverse consequences, such as over-valuing and relying excessively on online social networking sites (Liu and Ma, 2018), which may further increase depression (Wang et al., 2018). Some studies report that online peer support had no significant effect on depression among young people (e.g., Freeman et al., 2008; Ellis et al., 2011). Furthermore, a meta-analysis revealed that the impact of OSS in young population with mental-health issues was inconsistent and inconclusive (Ali et al., 2015). Therefore, more empirical research is necessary to identify the effect of OSS. Such insights will benefit the development of social support interventions.

### The present study

**Figure 1:**
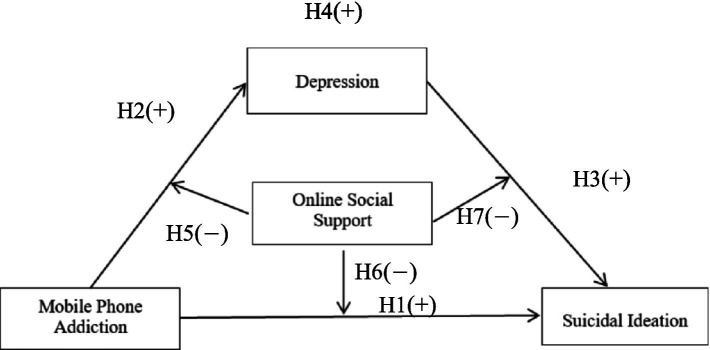
The proposed model.

We propose a model testing whether OSS would moderate the direct and indirect associations between MPA and suicidal ideation through depression (see Figure 1).

*H1*: MPA would be positively correlated with suicidal ideation.

*H2*: MPA would be positively correlated with depression.

*H3*: Depression would be positively correlated with suicidal ideation.

*H4*: Depression would mediate the relationship between MPA and suicidal ideation.

*H5*: OSS would moderate the correlation between MPA and depression. Specifically, the effect of MPA on depression would be weaker among individuals with higher (compared to lower) levels of OSS.

*H6*: OSS would moderate the correlation between MPA and suicidal ideation. Specifically, the effect of MPA on suicidal ideation would be weaker among individuals with higher (compared to lower) levels of OSS.

*H7*: OSS would moderate the correlation between depression and suicidal ideation. Specifically, the effect of depression on suicidal ideation would be weaker among individuals with higher (compared to lower) levels of OSS.

## Materials and methods

### Participants

Our target participants were Chinese university students. The inclusion criteria were (1) undergraduate students in college; (2) Chinese citizens; (3) use a mobile phone on daily base; (4) able to understand Chinese language.

In this study, we had eight variables (including three study variables: MPA, depression, online social support and five background variables: gender, grade, daily use time of mobile phone, single child and residence). *A priori* sample size estimation for multiple regression suggested that a minimum sample size of 962 individuals was required to detect a small effect size of.02 (α = 0.05, β = 0.90; Soper, 2011). A total of 1,123 college students, which was enough for our analysis, were therefore invited to participate in the present study.

### Procedures

Convenience sampling method was used. During October and November 2018, a closed cross-sectional survey was performed in classroom settings in a university in Wenzhou, China. The survey was traditional paper-pencil and self-administered. All eligible university students studying in major departments of the selected university (e.g., School of Stomatology, School of Psychiatric Medicine) were invited to participate and completed the survey. A psychology course lecturer assisted the researcher (a postgraduate student) with the data collection process and answered participants’ questions regarding the survey. The survey was anonymous and students have been ensured that there would not be negative consequence if they rejected or did not complete the survey. Informed consent of every participant was acquired at the beginning of the survey. The participants were assured confidentiality and that only our researchers (and not the course lecturer) could access the data. We provided the list of resources for information of seeking professional help at the end of consent form in case the participants needed it. All the questionnaires collected were sealed in an archive bag in front of all the participants and forwarded to the researcher for processing and carefully stored after data collection. A number of 1,123 college students were invited and 1,042 participants were included in the data analysis (81 cases were excluded as they did not complete the scales of the key study variables such as MPA and online social support), resulting in a response rate of 92.8%. Ethics Committee of the corresponding author’s institution had reviewed and approved the study.

### Measures

#### The mobile phone addiction index

Mobile Phone Addiction Index (MPAI; Leung, 2008) was administered to assess MPA. It consists of 17 items and measures four symptoms of MPA: unable to control cravings, loss feelings and anxiety, escaping and withdrawal, and impaired functioning. Participants were asked to rate the items (e.g., “You find that your engagement time on mobile phone is longer than expected”) on a 5-point Likert scale, ranging from 1(Never) to 5(Always). Higher score implies higher tendency of MPA. The MPAI has manifested adequate reliability and validity in Chinese university students (Lian et al., 2016). Cronbach’s α was.84 for this paper. The results of confirmatory factor analysis (χ^2^/df = 4.96, CFI = 0.922, TLI = 0.904, RMSEA = 0.062, SRMR = 0.053) showed that the MPA scale had good construct validity in the present study.

#### The center for epidemiologic studies depression scale

Chinese form of the 20-item Center for Epidemiologic Studies Depression Scale (CES-D-20; Chien and Cheng, 1985) was administered. It has displayed good validity in Chinese sample (Jiang et al., 2019). Students were asked to rate recurrence of items (e.g., “I feel sad”), with 0 to 3 (0 = *Rarely*; 1 = *Sometime*; 2 = *Occasionally*; 3 = *Most of the time*). Higher ratings imply greater depressive symptoms. In this investigation, Cronbach’s α of the scale was.91. The results of confirmatory factor analysis (χ^2^/df = 5.44, CFI = 0.922, TLI = 0.908, RMSEA = 0.065, SRMR = 0.049) showed that the CESD-20 had good construct validity in the present study.

#### College students’ online social support scale

College Students’ Online Social Support Scale (CSOSSS; Liang and Wei, 2008) was administered. The 23-item scale assesses four dimensions of OSS: social member/peer, emotional, information, instrumental. Sample items include, “When I feel lonely, I can talk to others through online social media” and “Posting my problems in online social media sites (such as QQ space, WeChat friends’ circle) can get a lot of responses.” Its 4-point Likert scale ranges from 1 (*Never*) to 4 (*Always*). Larger ratings reflect higher levels of OSS. The scale has been found with satisfactory reliability and validity (Liu et al., 2017). Cronbach’s α of this scale was.92. The results of confirmatory factor analysis (χ^2^/df = 5.72, CFI = 0.932, TLI = 0.922, RMSEA = 0.067, SRMR = 0.059) showed that the CSOSSS had good construct validity in the present study.

#### Self-rating idea of suicide scale

The 21-item Self-rating Idea of Suicide Scale (SIOSS, Xia et al., 2002) was used to measure suicidal ideation. It has been validated and shown good validity in Chinese population (Chen et al., 2013; Xie P. et al., 2018). These items cover three dimensions: sleep, desperation, and optimism. Sample items include, “Most of the time, I think it’s better to be dead” and “I want to end my life.” Each item was answered “yes” or “no” (0 = *No*; 1 = *Yes*). A higher total score implies higher suicidal ideation. The Cronbach’s α of SIOSS was.84. The results of confirmatory factor analysis (χ^2^/df = 3.14, CFI = 0.950, TLI = 0.944, RMSEA = 0.045, SRMR = 0.087) showed that the SIOSS had good construct validity in the present study.

### Statistical analyses

Descriptive statistics were computed for the independent variable (MPA), mediator (depression), moderator (OSS), and outcome variable (suicidal ideation). Spearman and Pearson correlation analyses examined the correlations among the background variables and psychological variables. The moderated mediation analyses were carried out with PROCESS 3.3 version (model 59; Hayes, 2013). Results of simple slope test were reported. This approach has been employed in many related studies (e.g., Xie X. et al., 2018). Bootstrapping analysis based on 5,000 resampling was conducted; for statistical significance, the 95% confidence interval (CI) must not include zero. The study variables (i.e., MPA, depression, OSS, and suicidal ideation) were standardized with Z-scores for the analyses. The 2% item-level missing data was dealt with using the expectation–maximization method (Graham, 2003; Dong and Peng, 2013). This method did not make any significant changes to the results of the correlation analyses and moderated mediation analyses compared to using the approach of excluding the incomplete questionnaires (data analysis supported this claim). Significant background variables such as gender, first year grade, and 8–24 h daily using time were included as potential covariates and controlled in the analysis. This method was commonly used in previous studies (e.g., Su et al., 2021; Jiang et al., 2022; Wang et al., 2022). IBM SPSS version 21.0. was utilized for these analyses. The significance value of p was.05.

## Results

### Demographic variables/characteristics

The demographic variables/characteristics of the 1,042 participants are presented in Table 1.

**Table 1:** Demographic characteristics of the participants (*n* = 1,042).

Variable	n	%
Gender		
Female	640	61.4%
Male	400	38.4%
Residence		
Rural	418	40.1%
Urban	595	57.1%
Single child		
No	489	46.9%
Yes	551	52.9%
Grade		
First year	299	28.7%
Second year	379	36.4%
Third year	360	34.5%
Forth year	4	0.4%
Major		
Preventive Medicine	225	21.6%
Rehabilitation	138	13.2%
Clinical Medicine	119	11.4%
Marketing	117	11.2%
Public Administration	88	8.4%
Psychology or Psychiatry	78	7.5%
General Medicine	58	5.6%
Oral Medicine	52	5.0%
Anesthesiology	50	4.8%
Forensic Medicine	44	4.2%
Other	73	7.0%
Hours of phone use per day		
Less than 4 h	277	26.6%
4.01–8 h	551	52.9%
8.01–24 h	178	17.1%

Those add up to less than 100% were because of missing values.

### Descriptive statistics and correlations among the background and study variables

The mean (M), standard deviation (SD), and correlations of the background and psychological variables are displayed (Table 2). MPA had a positive correlation with depression and suicidal ideation, respectively. Depression was positively associated with suicidal ideation. OSS was negatively correlated with depression and suicidal ideation, respectively.

**Table 2:** Descriptive statistics and correlation coefficients among covariates and study variables.

	n(%)/M(SD)	Max/Min	1		2		3		4		5		6	
			*r*	*p* value	*r*	*P* value	*r*	*P* value	*r*	*P* value	*r*	*P* value	*r*	*P* value
1. Female gender	640 (61.4%)	/	1											
2. First-year grade	299 (28.7%)	/	−0.11^***^	<0.001	1									
3. 8–24 h spent on MP daily	178 (17.1%)	/	0.02	0.62	−0.08^**^	0.01	1							
4. MPA	48.53 (10.74)	84/17	−0.06^*^	0.04	0.03	0.36	0.12^***^	<0.001	1					
5. Depression	15.92 (9.32)	58/0	0.01	0.70	−0.07^*^	0.03	0.08^**^	0.01	0.32^***^	<0.001	1			
6. OSS	62.50 (10.80)	92/23	−0.03	0.27	0.01	0.65	−0.03	0.29	0.13^***^	<0.001	−0.13^***^	<0.001	1	
7. Suicidal ideation	4.60 (3.90)	20/0	0.02	0.45	−0.06^*^	0.04	0.07^*^	0.04	0.20^***^	<0.001	0.72^***^	<0.001	−0.19^***^	<0.001

*n* = 1,042. MP = mobile phone. MPA = mobile phone addiction. OSS = online social support. Categorical variables (demographic variables) were dummy coded for Spearman correlation analyses. Pearson correlation analysis was conducted for the associations among study variables.

### Mediation effect analysis

Regression analysis showed that MPA was positively related with suicidal ideation while adjusting and not adjusting the covariates (*B* = 0.20, 95% CI: 0.14, 0.26, *p* < 0.001). The exclusion effect of control variables was 0.01. Results of mediation effect analysis showed that MPA had a positive association with depression (*B* = 0.31, 95% CI: 0.25, 0.37, *p* < 0.001), depression was positively related with suicidal ideation (*B* = 0.73, 95% CI: 0.69, 0.78, *p* < 0.001). The indirect effect of MPA on suicidal ideation *via* depression was significant (indirect effect = 0.23, 95% CI: 0.18, 0.28, *p* < 0.001), and the direct effect was not significant (*B* = −0.03, 95% CI: −0.08, 0.01, *p* > 0.05; Table 3). The changed R-square was 0.48, suggesting that the indirect effect explained 48% additional power. A full mediation effect was demonstrated.

**Table 3:** Mediation effect analyses for the association between mobile phone addiction and suicidal ideation *via* depression.

	Model 1 (Suicidal ideation)		Model 2 (Depression)		Model 3 (Suicidal ideation)	
	B	95%CI	B	95%CI	B	95%CI
Female gender	0.11	−0.02, 0.23	0.05	−0.07, 0.17	0.07	−0.02, 0.16
First year grade	−0.15	−0.29, −0.02	−0.16	−0.29, −0.03	−0.04	−0.13, 0.06
8–24 h spent on MP daily	0.11	−0.05, 0.27	0.07	−0.08, 0.23	0.06	−0.05, 0.17
MPA	0.20	0.14, 0.26	0.31	0.25, 0.37	−0.03	−0.08, 0.01
Depression					0.73	0.69, 0.78
*R ^2^*	0.05		0.11		0.53	
*P* value	<0.001		<0.001		<0.001	
*F*	13.16		29.59		224.29	

*p < 0.05, **p < 0.01, ***p < 0.001. MPA = mobile phone. MPA = mobile phone addiction.

### Moderated mediation effect analyses

The total effect of MPA on suicidal ideation was estimated after controlling for the covariates. As exhibited in Model 1 (see Table 4), MPA had a positive association with suicidal ideation (*B* = 0.22, 95% CI: 0.16, 0.28, *p < 0*.001), OSS was negatively related with suicidal ideation (*B* = −0.21, 95% CI: −0.27, −0.15, *p < 0*.001). The interaction effect of MPA and OSS on suicidal ideation was non-significant (*B* = 0.02, 95% CI: −0.03, 0.07, *p =* 0.370).

**Table 4:** Moderated mediation effect analyses for online social support.

	Model 1 (Suicidal ideation)		Model 2 (Depression)		Model 3 (Suicidal ideation)	
	*B*	*95%CI*	*B*	*95%CI*	*B*	*95%CI*
Female gender	0.10	−0.02, 0.22	0.05	−0.07, 0.17	0.05	−0.04, 0.13
First year grade	−0.15	−0.28, −0.02	−0.15	−0.28, −0.02	−0.03	−0.12, 0.06
8–24 h spent on MP daily	0.08	−0.07, 0.24	0.04	−0.11, 0.20	0.06	−0.05, 0.17
MPA	0.22	0.16, 0.28	0.34	0.28, 0.40	−0.02	−0.06, 0.03
Online social support	−0.21	−0.27, −0.15	−0.17	−0.23, −0.11	−0.09	−0.13, −0.04
MPA × Online social support	0.02	−0.03, 0.07	−0.03	−0.08, 0.02	0.06	0.02, 0.10
Depression					0.71	0.66, 0.75
Depression ×Online social support					−0.09	−0.13, −0.04
*R ^2^*	0.1		0.14		0.55	
*P* value	<0.001		<0.001		<0.001	
*F*	17.61		25.99		150.44	

MP = mobile phone. MPA = mobile phone addiction. Categorical variables were dummy coded for analysis.

**Figure 2:**
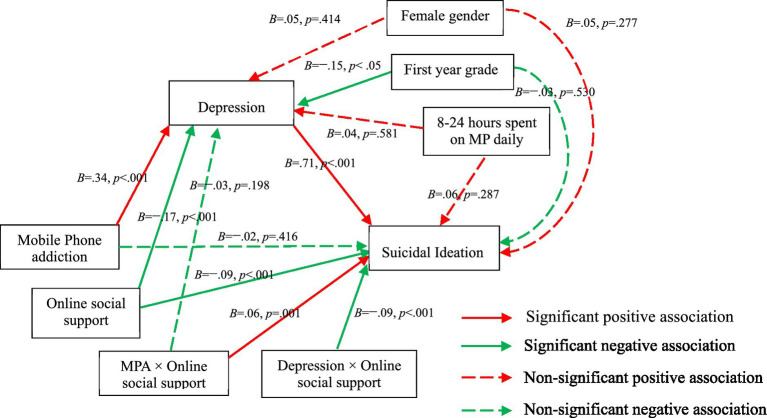
Statistical diagram for the moderated mediation model.

Then, the effects of MPA, OSS, and interaction effect of MPA and OSS on depression were examined after controlling for the covariates. As Model 2 displayed (see Table 4; Figure 2), MPA had a positive association with depression (*B* = 0.34, 95% CI: 0.28, 0.40, *p < 0*.001), OSS was negatively related with depression (*B* = −0.17, 95% CI: −0.23, −0.11, *p < 0*.001). A non-significant interaction effect of MPA and OSS on depression (*B* = −0.03, 95% CI: −0.08, 0.02, *p = 0*.198) was found, suggesting that the moderating effect of OSS in the association between MPA and depression was non-significant.

**Figure 3:**
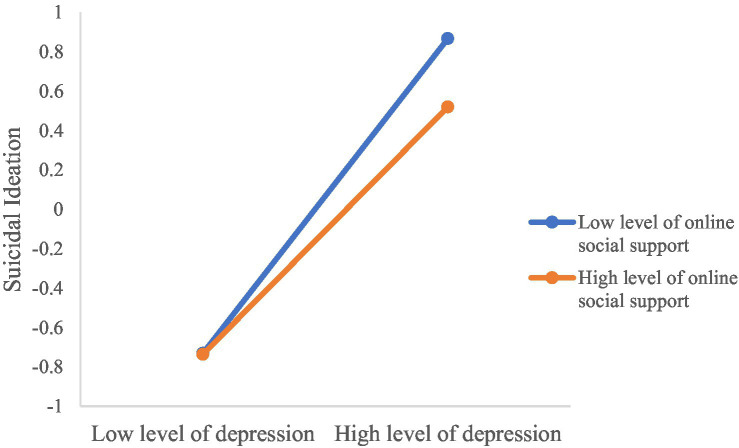
Simple slope test results for online social support moderating the association between depression and suicidal ideation. High = 1 standard deviations above the mean; Low = 1 standard deviations below the mean.

**Figure 4:**
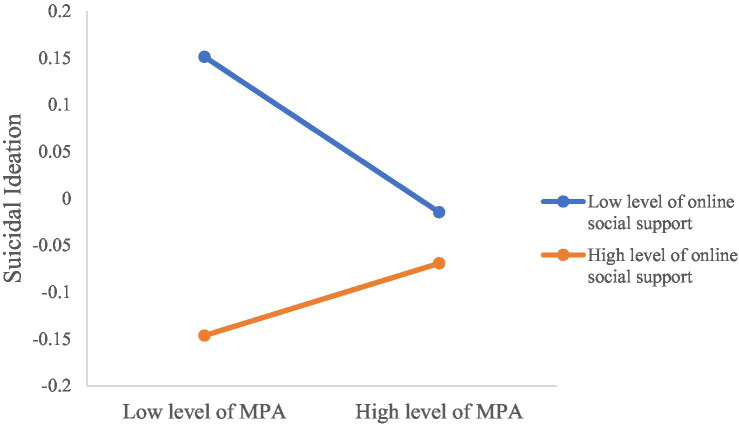
Simple slope test results for online social support moderating the association between mobile phone addiction (MPA) and suicidal ideation. High = 1 standard deviations above the mean; Low = 1 standard deviations below the mean.

Finally, MPA, OSS, the interaction of MPA and OSS, depression, the interaction of MPA and depression were entered as predictors while controlling for the covariates. In Model 3 (see Table 4; Figure 2), MPA had non-significant association with suicidal ideation (*B* = −0.02, 95% CI: −0.06, 0.03, *p = 0*.416). OSS was negatively related with suicidal ideation (*B* = −0.09, 95% CI: −0.13, −0.04, *p < 0*.001). The interaction effect of MPA and OSS was significant on suicidal ideation (*B* = 0.06, 95% CI: 0.02, 0.10*, p =* 0.001). Depression had a positive association with suicidal ideation (*B* = 0.71, 95% CI: 0.66, 0.75*, p < 0*.001). The interaction effect of depression and OSS was significant on suicidal ideation (*B* = − 0.09, 95% CI: −0.13, −0.04*, p < 0*.001). The moderation effects explained 2% additional power. Simple slope tests indicated that the positive correlation between depression and suicidal ideation was significantly weaker for university students who had high OSS (*B*_simple_ = 0.62, 95% CI: 0.56, 0.69, *p < 0*.001) than for those had low OSS (*B*_simple_ = 0.80, 95% CI: 0.74, 0.86, *p < 0*.001; Figure 3). For individuals with low OSS, MPA was negatively correlated with suicidal ideation (*B*_simple_ = − 0.08, 95% CI: −0.14, −0.02, *p =* 0.010; Figure 4), while for individuals with high OSS, the correlation between MPA and suicidal ideation become non-significant (*B*_simple_ = 0.04, 95% CI: −0.01, 0.10, *p =* 0.146).

## Discussion

This investigation is the first testing the moderation role of OSS in the association between MPA and suicidal ideation through depression among university students. The overall results support our hypotheses by showing that MPA was positively associated with suicidal ideation through increased depression, and this mediation model was moderated by OSS. The results can deepen our understanding of how MPA may increase university students’ suicidal ideation.

### The mediation role of depression

Firstly, H1 was supported. We found that MPA was positively linked with suicidal ideation in university students. It is novel to test this association among university students. The result is in compliance with past empirical research on adolescents (Wang et al., 2014; Chen et al., 2020). It highlights that suicidal ideation may be one of the potential negative consequences of problematic mobile phone use among university students. Furthermore, the results indicate that MPA was correlated with depression, which in turn was correlated with suicidal ideation, i.e., the association between MPA and suicidal ideation was mediated by depression, supporting H2-H4. This is consistent with both theoretical assumptions proposed by Hoge et al. (2017) and empirical studies on the roles of technology addiction (including MPA) in depression in young adults (Thomée et al., 2012; Ivanova et al., 2020). The finding echo the stress coping theory (Lazarus and Folkman, 1984) and the strain theory of suicide (Zhang, 2019) that MPA as a negative and maladaptive coping strategy may result in failure of (or inadequacy in) solving stressful situation, bring frustration and produce tortures and even suicidal ideation. Depression is a prevalent and significant emotional problem that is closely correlated with pain, hopelessness (Mee et al., 2011; Ribeiro et al., 2018) and suicidal ideation (Farabaugh et al., 2012; Nam et al., 2018). The positive correlation of depression and suicidal ideation further supports the three-step theory of suicide, which suggests that depression is highly associated with suicidal ideation (Klonsky and May, 2015). The mediation effect of depression highlights the substantial role of negative affective disorder in understanding the correlation between MPA and suicidal ideation. In other words, the influence of MPA on suicidal ideation was through the generation of depression. Suicidal ideation might occur only if a mobile phone addicted person develops depression. The finding highlights the significance of depression in predicting suicide for individuals with addictive behaviors, which is in line with previous literature (e.g., Yuodelis-Flores and Ries, 2015; Holman and Williams, 2022). Therefore, it is critical to reduce depression for those with MPA for suicide prevention. Intervention strategies such as mindfulness-based stress reduction, mindfulness-based cognitive therapy and cognitive-behavioral therapy are effective and can be utilized in the reduction of depressive symptoms (Butler et al., 2006; Hofmann and Gómez, 2017).

### The moderation role of online social support

To our best knowledge, this investigation is novel in examining the buffering effect of OSS in the relationships among MPA, depression and suicidal ideation. First, we found that it buffered the positive correlation between depression and suicidal ideation; namely, the correlation became weaker when OSS increased, supporting H7. This finding supports the buffering role of social support in mental-health problems (Cohen and Wills, 1985). Individuals with high OSS can obtain multiple facets of support such as emotional and informational support, thus influencing individuals’ coping and appraisal, which further functioning as a stable resource that protects people from mental health problems (Lakey and Cohen, 2000). The finding extends the understanding of Three-Step Theory by suggesting OSS as a buffer in the relationship between depression and suicidal ideation. OSS builds the sense of connectedness of the students with their families, friends and other social networks. Students with high OSS would perceive satisfying connectedness and thus would be less likely to have suicidal ideation when suffering from depressive symptoms such as psychological pain or hopelessness (Klonsky and May, 2015). The finding is in line with another study suggesting the protective role of OSS on depressive thoughts and feelings in university students (Cole et al., 2017). Furthermore, the finding of the buffering effect of OSS highlights the importance of exploring what people are doing on the phones and the relationship between specific activities and well-being in the future.

Interestingly, the association between MPA and suicidal ideation was non-significant among the participants with high OSS but significantly negative among those with low OSS. It may be because for those with low OSS and when the effect of depression was adjusted for, dependence of mobile phone may reduce their social anxiety and provide chance to meet their other basic psychological needs (e.g., reducing boredom, psychological pain and hopelessness) (Kardefelt-Winther, 2014; Casale and Fioravanti, 2015; Vansteenkiste et al., 2020; Rozgonjuk et al., 2021), thus decreasing their risk of suicidal ideation. Similarly, Jasso-Medrano et al. also found a significant negative association between social media addiction and suicidal ideation when depression was accounted for in university students (Jasso-Medrano and López-Rosales, 2018). The authors suggested that being addictive in social media could be protective against suicidal ideation when was related to depression (Jasso-Medrano and López-Rosales, 2018). MPA is often considered a way to escape from stress (Fu et al., 2020), and thus may have some short-term benefits. However, it is worth noting that, according to the simple slop tests, regardless of the levels of MPA, the overall levels of suicidal ideation among students with low levels of OSS were higher than those with high levels of OSS. The findings correspond with previous literature suggesting that disconnectedness (i.e., thwarted belonging) is just a facilitator of suicide while cannot explain the root cause of suicide (Van Orden et al., 2010). Qualitative interviews regarding the psychosocial needs and experiences of mobile phone users may help to better understand this result.

However, OSS did not significantly buffer the association between MPA and depression. It is inconsistent with some previous studies (Haden and Scarpa, 2008). A randomized controlled trial study also found that online peer support did not significantly reduce depression (Ellis et al., 2011). One plausible reason may be that social support is only effective for mild to moderate levels of stress (Ioannou et al., 2019) and so cannot buffer the adverse impact of high levels of stress that characterize MPA. Indeed, the symptoms of MPA, such as obsessive thoughts about mobile phones (craving) and anxiety when not using them (withdrawal), may induce great stress and are robust risk factors for depressive symptoms (Jun, 2016). More empirical studies of this moderating effect are needed before drawing any conclusion. Overall, although social support has been evidenced as a critical buffer and used in many situations (Yang et al., 2010; McGuire et al., 2018; Casale et al., 2019), the mitigation role of OSS is limited in the association between MPA and suicidal ideation *via* depression. Other potentially important moderators (e.g., perceived stress levels) should be explored in future studies.

### Implications

The findings have significant practical indications. Early detection of, and intervention in MPA and depressive symptoms may help to prevent suicidal ideation. Successful interventions for reducing MPA and depression are currently available. For example, cognitive-behavioral intervention based on mindfulness for MPA was found to be effective in alleviating MPA among university students (Lan et al., 2018). Antidepressant medications and cognitive behavioral-based interventions (e.g., stress coping and emotion regulation training) are widely used and effective for young people diagnosed with depression (Callahan et al., 2012). In addition, online support from friends, family members, important others, and community should be promoted as it is a well-documented protective factor against and buffer of suicidal ideation. Social support can be improved by traditional interventions such as nondirective supportive therapy and new interventions such as promoting social networks and support, which have proven to be successful in decreasing suicidal ideation and depression (Brent et al., 1997; Martin et al., 2011).

### Limitations and future directions

Firstly, selective bias may exist because of convenience sampling (for example, only 0.4% of the fourth-grade students were recruited as our participants). A cautious approach should be taken when generalizing the results to other college students. Second, cross-sectional design was employed; thus, causality among the studied variables could not be demonstrated. There may exist a reciprocal relationship among MPA, depression, and suicidal ideation. Longitudinal and experimental studies are needed for further evidence. Third, social desirability bias could be a problem as this study used self-report measures. Future studies should obtain information from multiple sources (e.g., peers, teachers) to validate participants’ responses. Forth, to better understand the role of social support, future work should test how different types of social support influence the association between MPA, depression, and suicidal ideation (e.g., online versus offline social support, support from important others versus non-important others/strangers). Future work should also test other moderators in addition to social support in the mediation model. Fifth, we did not investigate or include some important variables, such as age, parenting styles, history of childhood abuse, whether the students were mature students (i.e., students who have worked for a while and go back to study are facing more challenges and more likely to be depressed, English, 2018), as confounders. Future studies need to consider these potential factors. In addition, according to the ideation to action framework, (a) the development of suicide ideation and (b) the progression from suicide ideation to potentially lethal suicide attempt are separate processes with separate explanations and predictors. Therefore, whether these factors can prevent both suicidal ideation and suicide attempt should be tested by empirical studies.

## Conclusion

In summary, the present study investigated the mechanisms underlying the relationships between MPA and suicidal ideation. The moderated mediation model showed that depression mediated the association between MPA and suicidal ideation, and OSS moderated the associations between depression and suicidal ideation and between MPA and suicidal ideation. In other words, MPA may not directly have an impact on suicidal ideation, but rather, may influence suicidal ideation through generating depression. OSS may buffer the relationship between depression and suicidal ideation. The findings facilitate both researchers and mental health service providers to better understand how MPA may increase the risk of suicidal ideation. They also highlight the potential importance of and need for depression reduction and social support enhancement to prevent suicidal ideation among university students.

## Data availability statement

The data that support the findings of this study are available from the corresponding author, upon reasonable request.

## Ethics statement

The studies involving human participants were reviewed and approved by the Ethics Committee of the Wenzhou Medical University. The patients/participants provided their written informed consent to participate in this study.

## Author contributions

HH: formal analysis, writing – original draft, and writing – review and editing. XY and PM: conceptualization, validation, and writing – review and editing. CZ: investigation, data curation, validation, and writing-original draft preparation. BK: investigation, validation, and writing-original draft preparation. GZ: conceptualization, methodology, funding acquisition, data curation, and writing – review and editing. GL: conceptualization, methodology, funding acquisition, and writing – review and editing. All authors contributed to the article and approved the submitted version.

## Funding

This study was supported by the Wenzhou Medical and Health Projects (No. 2019B52).

## Conflict of interest

The authors declare that the research was conducted in the absence of any commercial or financial relationships that could be construed as a potential conflict of interest.

## Publisher’s note

All claims expressed in this article are solely those of the authors and do not necessarily represent those of their affiliated organizations, or those of the publisher, the editors and the reviewers. Any product that may be evaluated in this article, or claim that may be made by its manufacturer, is not guaranteed or endorsed by the publisher.

## Abbreviations

MPA: mobile phone addiction
OSS: online social support
